# Der gestielte myofasziokutane A. suralis medialis- oder lateralis Perforator - M. gastrocnemius-Lappen zur kniegelenksnahen Weichteilrekonstruktion

**DOI:** 10.1007/s00113-023-01303-2

**Published:** 2023-03-13

**Authors:** Thierry Schweizer, Andreas Gohritz, Steven John Lo, Dirk Schaefer, Rik Osinga

**Affiliations:** 1 Klinik für Plastische, Rekonstruktive, Ästhetische und Handchirurgie, Universitätsspital Basel, Spitalstr. 21, 4031 Basel, Schweiz; 2 Canniesburn Plastic Surgery Unit, Glasgow Royal Infirmary, 84 Castle Street, Glasgow, UK; 3 Zentrum für Muskuloskelettale Infektionen (ZMSI), Universitätsspital Basel, Spitalstr. 21, 4031 Basel, Schweiz; 4 Praxis beim Merian Iselin, Thannerstr. 80, 4054 Basel, Schweiz

## Einleitung

Der gestielte mediale Gastrocnemius-Muskellappen ist der Standardlappen für kniegelenknahe Weichteilrekonstruktionen. Hierbei kann die tendinöse Rückseite des Lappens verwendet werden, um Defekte der Kniegelenkkapsel/des Streckapparates zu rekonstruieren. Dabei wird das Grundprinzip angewendet, Gleiches mit Gleichem zu ersetzen. Liegt ein kompletter Riss der Quadrizeps- oder Patellarsehne vor, kann der Gastrocnemius-Muskellappen erweitert werden und ein Teil der Achillessehne mitgehoben werden, um die defekte Sehne zu rekonstruieren [1, 2]. Der Lappen kann auch mit einer Hautinsel gehoben werden, wenn das versorgende Perforatorgefäß miteingeschlossen wird. Speziell bei älteren Patienten kann aufgrund der schlafferen Haut eine größere Hautkomponente gehoben und der Hebedefekt trotzdem direkt verschlossen werden [3]. Beim Lappeneinbau kann die Hautinsel um den Perforator gedreht werden (Propellerlappen) [4]. Das myokutane Heben des chimären Lappens kann mit dem medialen oder lateralen M. gastrocnemius durchgeführt werden. Hierbei ist zu beachten, dass der muskulokutane Perforator über dem medialen M. gastrocnemius fast immer vorhanden ist, derjenige über dem lateralen M. gastrocnemius hingegen gelegentlich fehlt [5]. Offenbar besteht eine inverse Beziehung zwischen den oberflächlichen fasziokutanen Perforatoren, welche aus der A. suralis (den N. suralis begleitend) abgehen und den muskulokutanen Perforatoren (medial und lateral) aus dem tiefen System der A. suralis [6]. Die Morbidität der Spenderstelle (z. B. Sensibilitätsstörungen oder lokale Muskelschwäche) ist in beiden Fällen gering.

## Präoperative Vorbereitung

**Figure 1:**
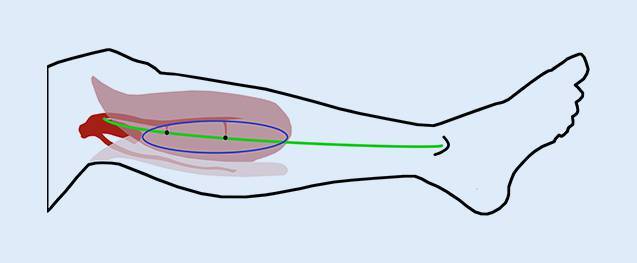

Die Gefäßversorgung des Standardlappens basiert auf der A. suralis medialis, die den medialen M. gastrocnemius versorgt und meist einen oder mehrere muskulokutane Äste (Perforatoren) in die Haut sendet (Abb. 1). Vor der Operation wird in Rückenlage und mit angewinkeltem Bein eine Linie (Abb. 1*grün*) von Mitte der Poplitealfalte zum medialem Malleolus gezeichnet, auf der mittels Handdoppler die beiden Perforatoren (Abb. 1*schwarze Punkte*) lokalisiert und eingezeichnet werden. Diese befinden sich rund 7 und 18 cm unterhalb der Poplitealfalte und entspringen meist im Bereich der Raphe, welche den medialen vom lateralen Anteil des M. gastrocnemius trennt [5]. Es besteht eine große Variabilität der Perforatorgefäße; diese sind häufig gewunden und korrelieren nicht immer direkt mit dem Dopplersignal [7, 8]. Die Hautinsel wird häufig in vertikaler, elliptischer Form (Abb. 1*blau*) um die Perforatoren gehoben, um möglichst einen Direktverschluss der Entnahmestelle zu ermöglichen.

## Operationsablauf

**Figure 2:**
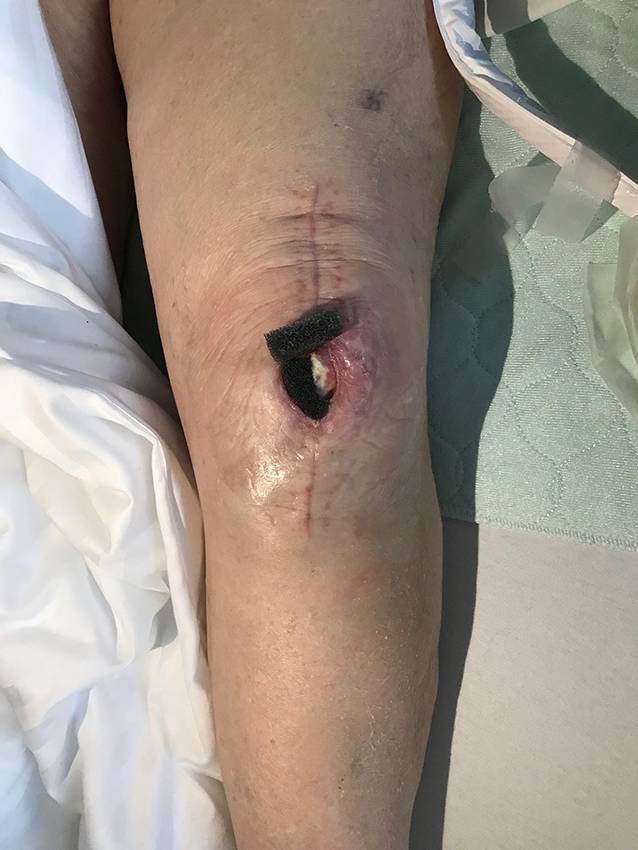

**Figure 3:**
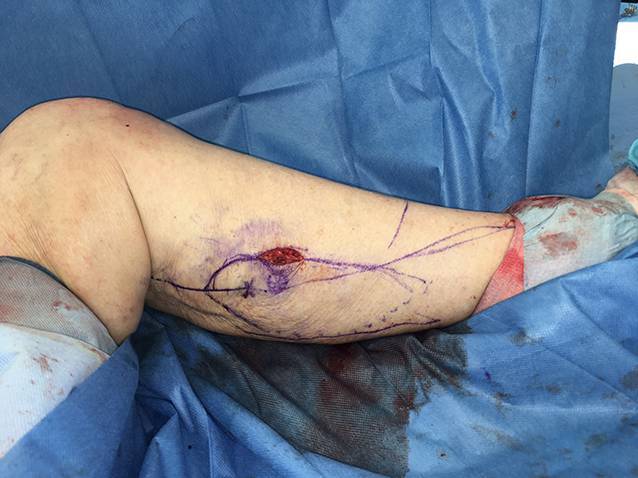

**Figure 4:**
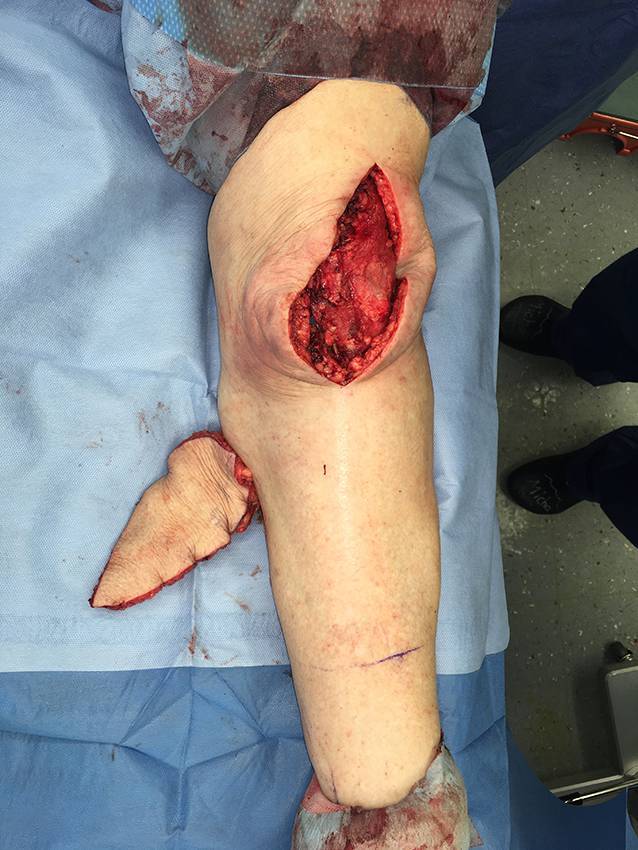

**Figure 5:**
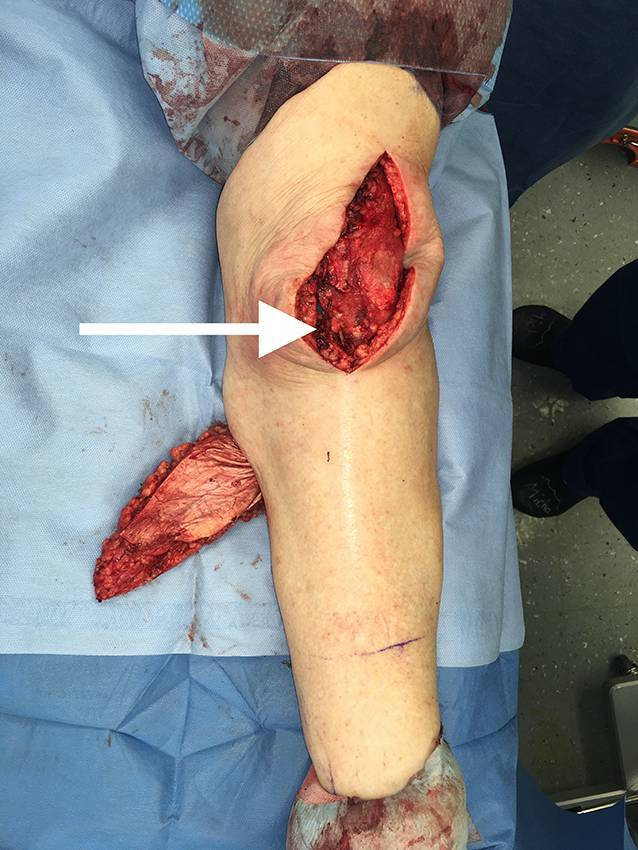

**Figure 6:**
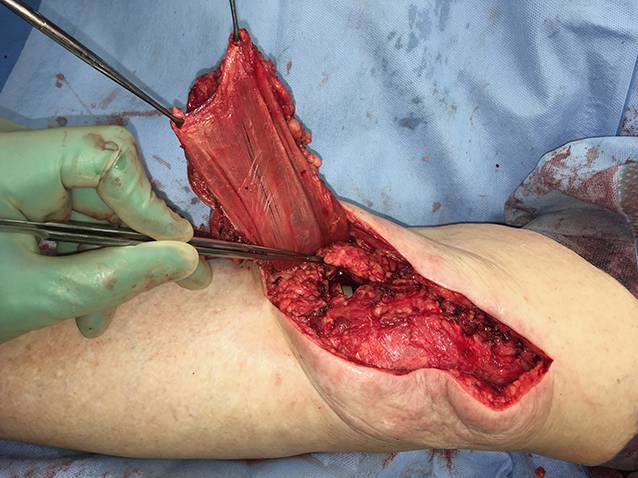

**Figure 7:**
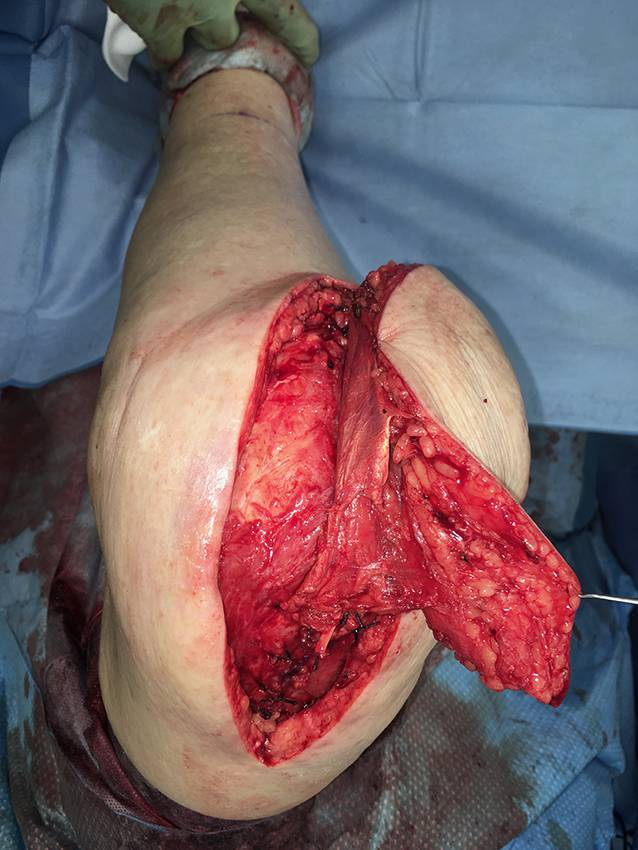

**Figure 8:**
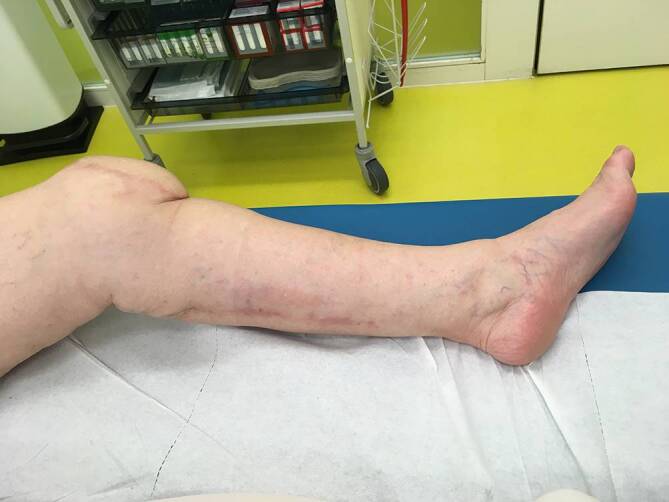

1. Die präoperative dreidimensionale Defektanalyse der Weichteile bestimmt die Rekonstruktion. Im gezeigten Beispiel besteht ein Hautdefekt, ein zusätzlicher Schaden des Streckapparates muss vermutet werden (Abb. 2). In Rückenlage und mit gebeugtem Knie werden die präoperativ identifizierten Perforatorgefäße mit dem Dopplergerät nochmals verifiziert und die geplante Hautinsel eingezeichnet. Eine Blutsperre ist nur selten indiziert.
2. Knapp anterior des distalsten Dopplersignals erfolgen die Hautinzision und die anschließende subfasziale Präparation für die Visualisierung und Bestätigung der Perforatoranatomie (Abb. 3). Je nach Befund kann die Planung der Hautinsel jetzt noch geändert werden.
3. Nun wird die Hautinsel komplett umschnitten und randständig mit Haltefäden am medialen M. gastrocnemius (MG) befestigt, um Zug auf die Perforatorgefäße durch das Eigengewicht der Haut zu vermeiden.
4. Von anterior her wird der MG vom M. soleus gelöst (diese Schicht zeichnet sich durch etwas Fettgewebe aus, wodurch die beiden Muskeln manuell relativ leicht voneinander gelöst werden können). In dorsale Richtung werden die Raphe und der darüber verlaufende N. suralis identifiziert und der mediale MG vom lateralen MG abpräpariert. Hierbei sollen der N. suralis sowie die V. saphena parva geschont und dem lateralen MG zugeschlagen werden.
5. Im Bereich des muskulotendinösen Übergangs wird der mediale MG distal abgesetzt (Abb. 4). Dabei kann die Hautinsel den Sehnenanteil überragen (Abb. 5). Je nach Verwendungszweck kann aber auch der tendinöse Anteil länger gewählt werden (z. B. bei lateralem Kapsel‑/Streckapparatdefekt). Unter Zug kann der mediale MG vom lateralen MG gegen proximal so weit abgelöst werden, bis der Lappen den Defekt ohne Spannung erreichen kann.
6. Stumpf kann nun subfaszial und möglichst nahe an der Tibia der Tunnel präpariert werden, durch den der MG gezogen werden soll. Im Bereich der Tibia wird die Faszie breit eröffnet. Zusätzlich soll die Unterschenkelfaszie quer inzidiert werden, um keine unnötige Spannung auf den Lappen (v. a. in Streckstellung) zu verursachen. Hierbei muss darauf geachtet werden, die V. saphena magna und den N. saphenus nicht zu verletzen. Der Tunnel soll genügend proximal sein, um den Lappen möglichst weit nach anterior (und wenn nötig proximal) positionieren zu können. Der Tunnel muss auch breit genug sein, um den Lappen nach Durchzug nicht durch die Faszienkante abzuknicken. Sollte der Lappen nicht genügend mobilisierbar sein, kann zusätzlich der Ursprung des Lappens an der medialen Femurkondyle gelöst werden. Hierbei muss auf eine penible Blutstillung, insbesondere der multiplen Venen des Lappens, geachtet werden, um ein Hämatom zu verhindern. Auch muss dann darauf geachtet werden, dass der gesamte Lappen dann nur am proximalen Gefäßstiel hängt und dass dieser nicht unter unnötigen Zug kommt.
7. Jetzt kann der Lappen durch den vorbereiteten Kanal hindurchgezogen werden. Dies ist in Streckstellung häufig einfacher zu bewältigen als in Beugestellung (Zusatzmaterial online: Video 1). Im Fallbeispiel zeigt sich schön der Kapseldefekt, welcher durch das tendinöse Gewebe rekonstruiert werden kann (Abb. 6).
8. Die Lappeneinpassung erfolgt idealerweise in 90°-Knie-Flexion, um eine Belastung der Weichteile bei der postoperativen Mobilisation des Knies zu vermeiden (Abb. 7). Der tendinöse Lappenanteil ist ideal, um den Lappen beim Einnähen in Position zu halten.
9. Die Entnahmestelle kann bis zu einer Breite von 7–8 cm meist direkt verschlossen werden (Abb. 8, 3 Monate postoperativ). Eine Redon-Einlage sowohl im Bereich des rekonstruierten Anteils wie auch im Bereich der Entnahmestelle ist fakultativ.

## Nachbehandlung

Der Patient soll während mindestens einer Woche mittels Knieorthese das Knie in Streckstellung horizontal lagern bei gelockerter Bettruhe. Danach erfolgt im Fall einer Knietotalprothese unter Vollbelastung das Lappentraining, dies weiterhin in Streckstellung in Knieorthese. Im Bett kann das Knie nach 2 Wochen ohne Belastung sukzessive zuerst passiv, danach aktiv unter physiotherapeutischer Anleitung mobilisiert werden, je nach Zustand der Weichteile (Wundheilungsstörungen, Lappendurchblutung). Die Intensität des Lappentrainings richtet sich dabei nach der Reaktion der Lappendurchblutung auf die Mobilisation (venöse Stauung?). Das gesamte Bein soll während der gesamten Rehabilitation elastisch gewickelt und im Verlauf ein Kompressionsstrumpf der Klasse II angepasst werden (Zusatzmaterial online: Video 2).

## Bemerkungen

**Figure 9:**
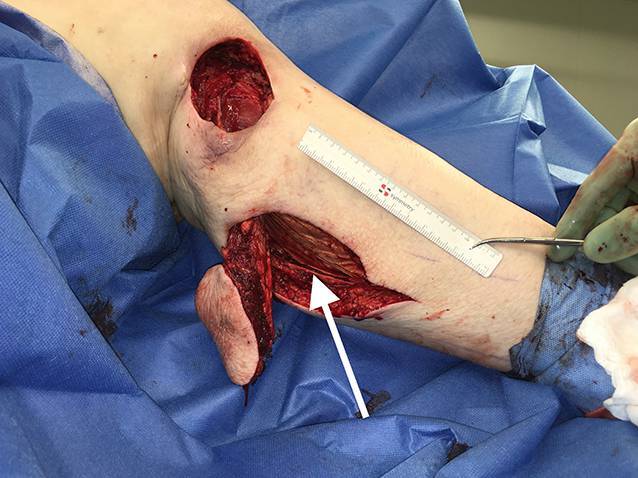

Die beschriebene Operationstechnik kann vom medialen auch auf den lateralen MG übertragen werden. Hierbei muss beachtet werden, dass der N. suralis durch das Lappengebiet läuft und geschont werden sollte, und dass der laterale MG häufig einen etwas kürzeren Muskelbauch hat sowie zusätzlich für die Defektrekonstruktion im anterioren Knie- und Unterschenkelbereich eine längere Wegstrecke benötigt, da der gestielte Lappen zusätzlich den Weg um die Fibula zurücklegen muss (*Cave* N. peronaeus communis – Neurapraxie durch Druck). Für kombinierte Kapsel-Weichteil-Defekte ist der gestielte myokutane laterale MG ideal (Abb. 9, 10 und 11).

**Figure 10:**
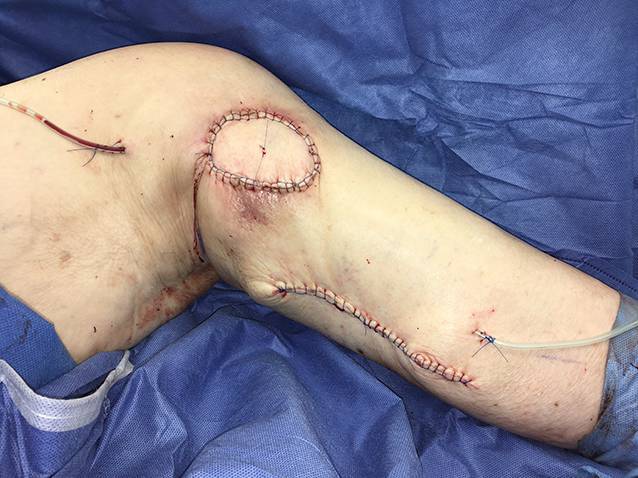

**Figure 11:**
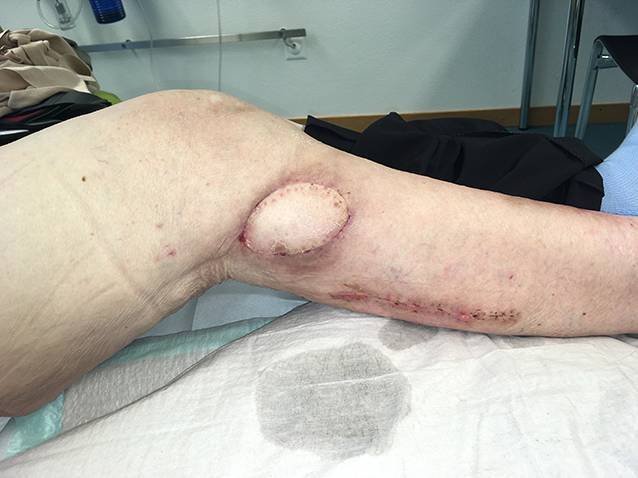

Bei einem zweizeitigen orthoplastischen Vorgehen sollte die Weichteilrekonstruktion möglichst früh erfolgen. Bei implantatassoziiertem Infekt nach Knietotalprothese z. B. während der gleichen Operation wie die Knie-TP-Explantation und Spacer-Einlage. Der Lappen kann nach einigen Wochen problemlos wieder über den gleichen Zugang gehoben, der Spacer entfernt, eine neue Totalprothese implantiert und der Lappen wieder eingepasst werden.

## Supplementary Information





